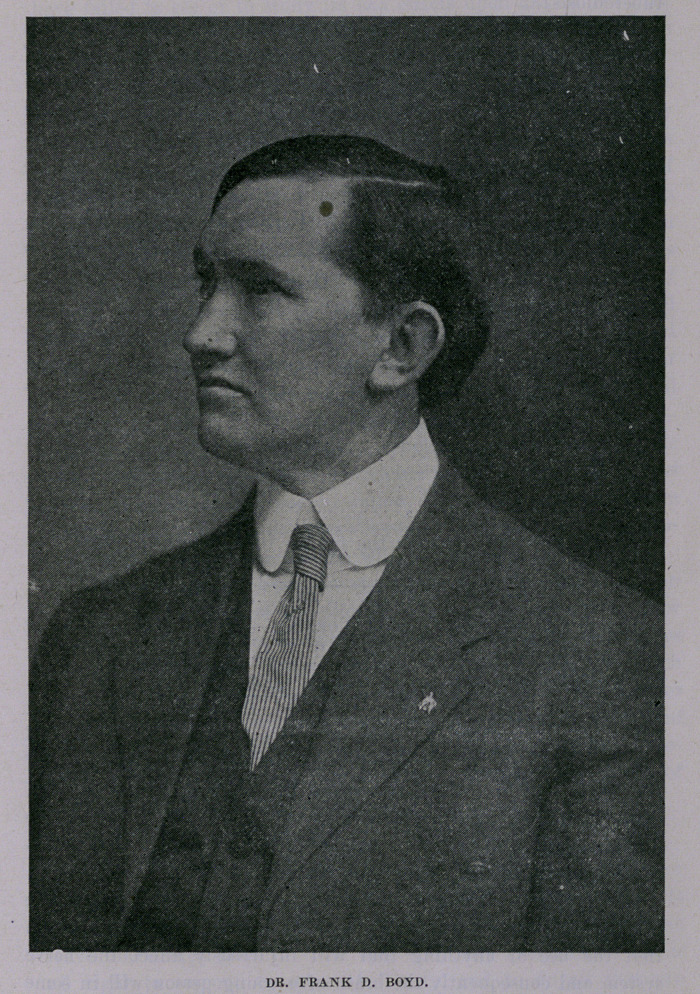# Dr. Frank D. Boyd, President Texas Medical Association

**Published:** 1914-06

**Authors:** 


DR. FRANK D. BOYD, PRESIDENT TEXAS MEDICAL ASSOCIATION.—Dr. Frank Douglas Boyd, the new President of the Texas Medical Association, is a son of John A. and Amy E. Boyd, and was born at Rusk, Texas, December 24, 1867.

He was educated in the public schools in Rusk and graduated from the Masonic Institute. Later he attended the Agricultural and Mechanical College at Bryan, Texas, and in 1890 graduated from the Medical Department of the University of Louisville. His record in that school was such that he was elected as assistant to the Chair of Eye, Ear, Nose and Throat, under Dr. William Cheatham, and remained there for about two years. In 1892 he was married to Miss Mattie E. Callahan, of Louisville, Ky., and soon after located at San Antonio, Texas, where he remained for about four years, going from there to Chicago, where he was associated as assistant with Dr. E. Fletcher Ingalls. He remained there only about six months, returning then to his native State and locating in Fort Worth, where he has continuously since devoted his entire attention to diseases of the eye, ear, throat and nose.

Dr. Boyd is Professor of Laryngology in the Medical Department of Texas Christian University. He has always been active in the medical affairs of the State, as well as those of the nation.

As a Fellow of the World's Congress of Medicine, having attended the last meeting in London, a Fellow of the International Congress of Otology, a Fellow of the American Academy of Ophthalmology and Oto-Laryngology, a Fellow of the American Medical Association, an honorary member of the Oklahoma Medical Society, and a member of many county and district medical societies of Texas, he has been conspicuously prominent in those things that make for the advancement of the profession.

Dr. Boyd has contributed many scientific articles and addresses to national, State and county publications and before medical societies and associations, some of which have attracted nation-wide attention. While always busily engaged professionally he is active in the municipal affairs of his home city, is a deacon in the Broadway Baptist Church, is a Shriner and a Rotarian.

The Texas Medical Association is to be congratulated on the election of Dr. Boyd to the Presidency, for he is a diligent student, a tireless worker, a man of pleasing address and strong personality, and is thoroughly in love with the profession to which

DR. FRANK D. BOYD.

he has devoted his life. The TEXAS MEDICAL JOURNAL predicts a year of unusual achievements for the Association under Dr. Boyd's splendid leadership.

THE *New York Weekly Bulletin of Health* makes the following interesting statement in regard to Dr. Friedmann's treatment of tuberculosis:

"Requests for information concerning the effect of the so-called Friedmann tuberculosis treatment, prompted the Department of Health to ascertain the recent status of seventy-seven cases, treated in hospitals in this city at the time of Dr. Friedmann's dramatic and widely advertised visit.

Of the total of seventy-seven patients, nineteen could not be found, while eleven were reported to have moved out of town permanently, so that nothing could be learned of their present condition. The Department was therefore able to obtain reports on but forty-seven of the seventy-seven cases in question. The reports are summarized as follows:

| | |
|---|---|
| At home | 5 |
| In hospitals and sanatoria (indicating failure to cure) | 22 |
| Attending clinics (showing need of further treatment) | 7 |
| Attended by private physician | 1 |
| Died | 12 |
| Total | 47 |

Comment is unnecessary; the figures tell their own story."

## Figures and Tables

**Figure f1:**